# Improving Antioxidant Capacity in Children With Autism: A Randomized, Double-Blind Controlled Study With Cysteine-Rich Whey Protein

**DOI:** 10.3389/fpsyt.2021.669089

**Published:** 2021-09-30

**Authors:** Ana Maria Castejon, Jordan Ashley Spaw, Irina Rozenfeld, Nurit Sheinberg, Susan Kabot, Alexander Shaw, Patrick Hardigan, Rogerio Faillace, Edward E. Packer

**Affiliations:** ^1^Department of Pharmaceutical Sciences, College of Pharmacy, Nova Southeastern University, Fort Lauderdale, FL, United States; ^2^Center for Collaborative Research, Institute for Neuro Immune Medicine, Nova Southeastern University, Fort Lauderdale, FL, United States; ^3^Mailman Segal Center, Nova Southeastern University, Fort Lauderdale, FL, United States; ^4^Statistical Consulting Center, College of Osteopathic Medicine, Nova Southeastern University, Fort Lauderdale, FL, United States; ^5^Department of Pediatrics, College of Osteopathic Medicine, Nova Southeastern University, Fort Lauderdale, FL, United States

**Keywords:** autism spectrum disorders, cysteine-rich whey protein, VABS-II, GSH, complementary and alternative medicine, oxidative stress

## Abstract

Previous studies indicate that children with autism spectrum disorder (ASD) have lower levels of glutathione. Nutritional interventions aim to increase glutathione levels suggest a positive effect on ASD behaviors, but findings are mixed or non-significant. A commercially available nutritional supplement comprising a cysteine-rich whey protein isolate (CRWP), a potent precursor of glutathione, was previously found to be safe and effective at raising glutathione in several conditions associated with low antioxidant capacity. Therefore, we investigated the effectiveness of a 90-day CRWP intervention in children with ASD and examined whether intracellular reduced and oxidized glutathione improvements correlated with behavioral changes. We enrolled 46 (of 81 screened) 3–5-year-old preschool children with confirmed ASD. Using a double-blind, randomized, placebo-controlled design, we evaluated the effectiveness of daily CRWP (powder form: 0.5 g/kg for children <20 kg or a 10-g dose for those >20 kg), compared with placebo (rice protein mimicking the protein load in the intervention group), on glutathione levels and ASD behaviors assessed using different behavioral scales such as Childhood Autism Rated Scale, Preschool Language Scale, Social Communication Questionnaire, Childhood Behavioral Checklist and the parent-rated Vineland Adaptive Behavior Scale, 2nd edition (VABS-II). Forty children (CRWP, 21; placebo, 19) completed the 90-day treatment period. Improvements observed in some behavioral scales were comparable. However, the VABS-II behavioral assessment, demonstrated significant changes only in children receiving CRWP compared to those observed in the placebo group in the composite score (effect size 0.98; 95% confidence intervals 1.42–4.02; *p* = 0.03). Further, several VABS-II domain scores such as adaptive behavior (*p* = 0.03), socialization (*p* = 0.03), maladaptive behavior (*p* = 0.04) and internalizing behavior (*p* = 0.02) also indicated significant changes. Children assigned to the CRWP group showed significant increases in glutathione levels (*p* = 0.04) compared to those in the placebo group. A subanalysis of the VABS-II scale results comparing responders (>1 SD change from baseline to follow up) and non-responders in the CRWP group identified older age and higher levels of total and reduced glutathione as factors associated with a response. CRWP nutritional intervention in children with ASD significantly improved both glutathione levels and some behaviors associated with ASD. Further studies are needed to confirm these results.

**Clinical Trial Registration:** https://clinicaltrials.gov/ct2/show/study/NCT01366859, identifier: NCT01366859.

## Introduction

Autism is a complex neurodevelopmental disorder that affects 1 in 54 children in the United States, with four times as many males diagnosed than females (1). Autism was first standardized as a mental disorder in 1980 (2), but its exact etiology remains undetermined. The spectrum of impairments noted in this disorder have coined the umbrella term “Autism Spectrum Disorder” (ASD). The fifth edition of the Diagnostic and Statistical Manual of Mental Disorders (DSM-V) defines ASD as having “deficits in social interaction and communication together with restricted and repetitive behaviors and interests” (American Psychiatric Association 2013) (3). Clinical symptoms are used to diagnose children with ASD around onset at age 3 years. A myriad of behavioral assessments are utilized for diagnostic purposes, with the Autism Diagnostic Observation Schedule (ADOS) (4, 5) as the gold standard. Despite extensive research, no definite biomarker for diagnosis or treatment has been detected (6). Early intervention programs and special schooling are the most effective for those with this neurodevelopmental disorder, and although outcomes of early intervention vary, all children benefit (7). A combination of applied behavioral analysis along with other educational, developmental, occupational and speech therapies are commonly applied to affected children with limited results (8). Thus, the need for other effective treatments for core symptoms of ASD is dire.

The heterogeneity of ASD can be observed in the neurologic, metabolic, and immunologic systems, etc. (9, 10). Therefore, it is speculated to be a multi-factorial disorder, involving epigenetics, genetics, and environmental factors. Oxidative stress may serve as a link between the different systems affected in this condition (10). Oxidative stress occurs when there is an imbalance between reactive oxygen species (ROS) and/or reactive nitrogen species and antioxidant capacity. Glutathione, the major endogenous antioxidant, is the body's primary defense against damage from ROS and is low in those with ASD (11–20).

Metabolites in the transmethylation and transsulfuration pathways, responsible for glutathione production are imbalanced in those with this disorder. Children with ASD also have significant decreases in methionine levels and the ratio of plasma S-adenosylmethionine (SAM) to S-adenosylhomocysteine (SAH) (SAM: SAH ratio), an index of methylation capacity (16, 21, 22).

Most importantly, children with ASD also have decreased levels of total glutathione and the reduced or active form of glutathione (16, 17). Cysteine, the rate limiting amino acid in glutathione synthesis, is significantly decreased in ASD relative to control children, suggesting that glutathione synthesis is insufficient to maintain redox homeostasis (16). These significant decreases in total and free plasma glutathione and the redox ratio (active reduced: inactive oxidized glutathione) in children with ASD is of particular concern due to the importance of this system for normal cell function.

Although prior nutritional interventions addressing antioxidant capacity have successfully improved glutathione levels, the association between these changes and autistic behavior has been less compelling. For example, N-acetylcysteine (NAC), which has a similar mechanism of action to the supplement utilized in this study, was effective at improving irritability in children with ASD (23). However, in a more recent study using NAC, glutathione production was increased but there was no significant improvement in social skills in youth with ASD (24). Other supplements, such as methylcobalamin, folic acid, folinic acid, sapropterin (a synthetic form of tetrahydrobiopterin), and combination treatments have also been investigated to improve antioxidant capacity and/or ASD behaviors based on abnormalities in the transmethylation/transsulfuration pathways and their effectiveness seems promising (25–30). Omega 3 fatty acids, vitamin C, vitamin D, and sulforaphane have also been studied in ASD, targeting oxidative stress via different mechanisms of action with varying results (25, 31–35). Other dietary interventions including essential fatty acids, carnitine, digestive enzymes, and a gluten-free, casein-free, soy-free diet, have also shown benefit in improving non-verbal IQ and ASD symptoms, in children with ASD over a year (36). Given that pharmacological alternatives only targeting specific comorbid conditions are limited, and with a significant burden of side effects, further studies utilizing complementary and alternative medicine for the treatment of ASD are justified.

A nutritional supplement composed of a cysteine-rich whey protein isolate (CRWP) that serves as a potent glutathione precursor, Immunocal®, is commercially available. Specific proteins in this supplement such as lactoferrin, serum albumin, alpha-lactalbumin, and immunoglobulins, are rich in cysteine and cystine residues, which are bioavailable for cellular absorption and subsequent glutathione synthesis. In prior clinical trials, CRWP was able to raise glutathione levels in those with obstructive lung disease (37), liver dysfunction in patients with chronic hepatitis B (38), cystic fibrosis (39), and healthy athletes (40, 41). CRWP was found to be safe and tolerable in a 6-week open-label study in children with ASD, demonstrating a trend toward an improvement in autistic behaviors (40).

In this study, we used a double-blind placebo-controlled design to explore the effectiveness of a 90-day intervention with a nutritional supplement containing CRWP in treating ASD core behavioral symptoms and elevating glutathione levels in 3–5-year-old preschool children with ASD. We also investigated whether improvements in intracellular glutathione correlated with behavioral changes. A comprehensive and age-specific behavioral assessment battery utilizing a total of eight different tests was used to assess which core areas of ASD could be impacted by this intervention.

## Materials and Methods

This study adhered to CONSORT guidelines and was approved by the Institutional Review Board at Nova Southeastern University in Fort Lauderdale, FL, USA. It was registered at the US National Institutes of Health (ClinicalTrials.gov) #NCT01366859. Parents of participants provided written informed consent (visit 1). This double-blind placebo-controlled study was performed between May 2011 and October 2016 at Nova Southeastern University (Fort Lauderdale, FL, USA) at three locations: The Mailman Segal Center (informed consent and behavioral assessments: visits 1, 3, 4, 5, 6, and 7), Nova Southeastern University Clinic (pediatric visits 2 and 8, including blood sampling), and the blood samples were quantified at the College of Pharmacy.

### Study Population

Participants were recruited from South Florida by the Mailman Segal Center using snowball sampling, in which additional participants are recruited by hearing about the study from initial participants. Initially, the ASD diagnoses were self-reported by the parents; however, they were confirmed after inclusion in the study according to the Diagnostic Statistical Manual (DSM-IV and DSM-V) criteria assessed by clinical psychologists using the ADOS and Autism Diagnostic Interview-Revised (ADI-R) test at baseline. Inclusion criteria included that the participants have ASD and be within 3–5 years of age at the start of, and during the trial period.

Exclusion criteria included: (i) allergies to milk, rice, or nuts; (ii) major medical problems, including cardiac, liver, endocrine, or renal disease; (iii) history of seizure disorder or gross neurological deficit; (iv) concomitant treatment with psychiatric medication; (v) current diet supplementation with N-acetylcysteine, alpha-lipoic acid, whey protein or higher than regular multivitamin doses of vitamin B12 or folic acid; (vi) comorbid diagnosis: Fragile X syndrome, tuberous sclerosis, phenylketonuria or fetal alcohol syndrome or (vii) acute illness. The comorbid conditions of Fragile X syndrome, tuberous sclerosis, phenylketonuria, or fetal alcohol syndrome were excluded because these children present some autistic behavioral features, but the origin is known. Genetic tests were not performed by the investigators to confirm exclusion diagnostics. However, the children's pediatricians confirmed exclusion and inclusion criteria by completing the Pediatrician Form, which also provided details of general health and previous medical history and recommendation for participation in the trial. Participants in both groups were permitted to continue taking multi-vitamins, probiotics, and other medications/supplements as long as they were not those mentioned in the exclusion criteria (i.e., known to significantly raise glutathione levels).

### Study Visits

**Figure 1:**
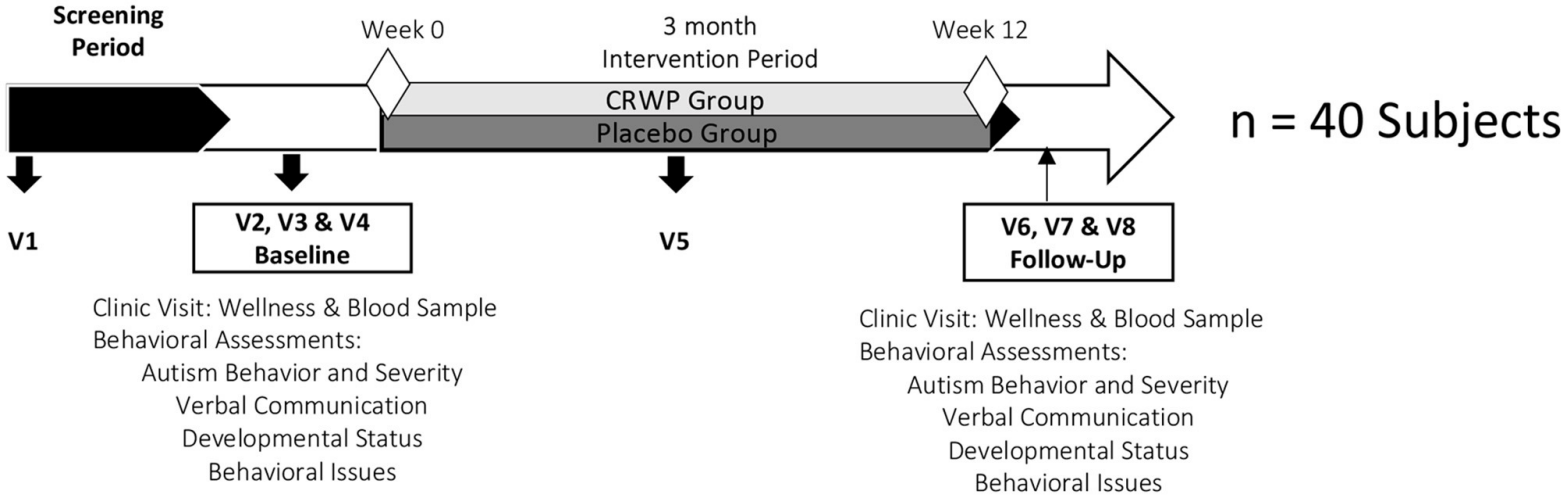
Study timeline. CRWP, cysteine-rich whey protein; V1-V8, Study visits.

The timeline of the study is shown in Figure 1. Visit 1 (Mailman Segal Center) consisted of an initial assessment of inclusion and exclusion criteria and the informed consent process. Only after parents agreed to enroll their children in the study and signed informed consent, a detailed medical history, current drug/supplement intake, and some demographic information about the child and parents were collected. Pediatrician forms, to be completed by the children's pediatricians, were also provided to the parent/caregivers at this visit. The objective of this form was to confirm inclusion/exclusion criteria with the child's pediatrician and inform the physician about his/her participation in a clinical trial.

Visit 2 (Nova Southeastern University Clinic) was scheduled after the parents returned the Pediatrician Form. This appointment consisted of a wellness exam conducted by Nova Southeastern University pediatricians to confirm that the child was otherwise healthy. A blood sample was obtained at this visit to assess reduced, oxidized and total glutathione as oxidative stress biomarkers. Separate samples were collected to assess liver and kidney function as well as cell blood count and were submitted to an independent laboratory (Quest Diagnostic™, Davie, FL). The pediatricians participating in this study could confirm continuation, or not, into the study after the clinical laboratory results were available.

Visits 3 and 4 for baseline measurements (Mailman Segal Center) consisted of the behavioral assessments conducted by Nova Southeastern University clinical psychologists. These consecutive visits were conducted no later than 15 days after visit 2. All eight behavioral assessments (see below) were split between these two visits, lasting an average of 2 h each. When needed, a third appointment was scheduled to avoid overwhelming the child with excessive testing and to minimize evaluation errors. These two visits were scheduled within a 15-day window. At the end of visit 4, children were randomized to either placebo (rice protein) or intervention (CRWP) and parents were given a diary to measure adverse effects or unusual events, together with the canister containing the study product in powder form with a measuring scoop. Parents and caregivers were asked to contact the principal investigator or study personnel if there were any questions or appearance of suspected side effects.

Visit 5 (Mailman Segal Center) was scheduled between weeks 6 and 7 (middle visit) to collect the remaining powder in the canister and parents' diaries and to provide new canisters for the next period. At this visit, a $25 Walmart gift card was provided to parents as compensation. The primary purpose of this visit was to assess compliance and record adverse events.

Visits 6 and 7 for follow-up assessment at week 12 were conducted as indicated for baseline visits 3 and 4. The same clinical psychologists performed the behavioral tests in the same sequence as baseline. Parents were instructed to continue with the study product daily dose until the next and final visit 8.

Visit 8, the follow-up/final visit (Nova Southeastern University Clinic), was scheduled no later than seven days after visit 7 (weeks 12–13). Visit 8 was performed as stated previously for visit 2. The final canister and parents' diaries were collected at this time. An additional $25 Walmart gift card was given to parents after completing this visit.

All families whose children participated and completed the 90-days trial period received a signed form by the principal investigator with instructions about how to contact the manufacturer of CRWP (Immunocal®, Immunotec Inc., Montreal, Canada) to obtain the supplement quarterly at no extra cost for up to 3 years (this was voluntary if the parent signed and gave consent).

### Intervention and Placebo

The intervention group received CRWP, commercially available as Immunocal® and provided by Immunotec® Inc. It should be noted that Immunocal® is included in the Physician's Desk Reference (42). Rice protein was used as placebo to mimic the protein load in the intervention group and was obtained from Thera-Plantes Inc. (Montreal, Canada). Both, CRWP (Immunocal®) and placebo (rice protein) treatments were provided to parents and caregivers in powder form in unlabeled canisters. A daily dose of 0.5 g/kg for children under 20 kg or a 10-g dose for those over 20 kg was taken by children in both arms of the study for at least 90 days. Clear instructions were given to parents and caregivers on how to reconstitute the powders using liquids and/or foods avoiding the use of a blender or heat. Parents were told how to measure the dose using measuring spoons provided by study personnel. The child's guardian was given the CRWP or the placebo randomized in powder form at two different visits. The first half of the product required for the study (with some excess) was given in a canister at visit 4. At visit 5, when returning the diaries, the canister was also returned for weighing to check compliance, and the other half of the study product was provided in a new canister for the remainder of the study. These canisters were then returned at week 12 (follow-up), and the weight of the canister and remaining product and entries in the parent's diary form were used to assess compliance.

Simple (1:1) randomization was performed using a randomization website by the blinded clinical research coordinator. Unique identifiers (randomization numbers matching canister numbers) were created to correspond with each participant in the trial. Additionally, the blinded clinical research coordinator also completed allocation concealment, which was only shared with the director of clinical research at the institution and who did not participate in the study. Trial investigators and participants were unaware of patients' allocation into groups during the trial. All study staff, participants, and parents/legal guardians were blinded to treatment group assignment.

### Outcome Measurements

All primary (behavioral measurements) and secondary outcomes (intracellular glutathione levels and adverse events) were obtained at baseline and study end. The diaries given to parents at visits 4 and 5 to record any side effects and/or unusual events were requested to be returned after completing the study.

#### Behavioral Assessments

Behavioral analysis in areas of ASD behavior and severity, communication, developmental status, and behavioral problems were conducted at baseline visits 3 and 4 as well as at the end of the study during follow-up visits 6 and 7. Trained assessors administered the battery of assessments, eight different tests in total, over a consecutive 2-day period. Assessors achieved reliability with each other before beginning the assessment process to minimize sources of error; the same assessor was responsible for administering the entire battery for a participant (baseline and follow-up). Additionally, the behavioral assessment teams were blind to each other's results.

Three behavioral assessments were performed in the ASD behaviors and severity domain: (1) ADOS, (2) Childhood Autism Rating Scale (CARS), and (3) the ADI-R. The ADOS and the ADI-R were utilized solely as inclusion criteria measurements. The ADOS is a semi-structured assessment administered by a trained assessor that consists of various activities that allow the observation of social and communication behaviors related to the diagnosis of ASD (5). In this study, participants were either given Module 1 or 2. Module 1 is intended for those who do not consistently use phrase speech, while Module 2 is used for those who use phrase speech but are not verbally fluent. The CARS is a 15-item behavior rating scale used to identify children with ASD and distinguish the severity of the disorder (43). The ADI-R is a comprehensive interview administered to parents that provides a thorough assessment of individuals with ASD (44). It focuses on three functional domains: Language/ Communication, Reciprocal Social Interactions, and Restricted/Repetitive and Stereotyped Behaviors and Interests.

Communication was assessed using the Social Communication Questionnaire (SCQ) and the Preschool Language Scale 5th Edition (PLS-5). The PLS-5 provides a comprehensive assessment of children's receptive and expressive vocabulary and is administrated by a trained assessor (45). The SCQ is a brief instrument that evaluates communication skills and social functioning in children with ASD (46) and is completed by the child's primary caregiver.

The developmental status of each participant was measured using the Mullen Scales of Early Learning (MSEL) administered by a trained assessor and the Vineland Adaptive Behavior Scale, 2nd edition (VABS-II) completed by parent interview. The MSEL is a developmentally integrated system that assesses language, motor, and perceptual abilities for children aged from birth to 68 months of age (47). It contains five scales: Gross Motor, Visual Reception, Fine Motor, Expressive Language, and Receptive Language. This assessment identifies a child's strengths and weaknesses and assesses early intellectual development and readiness for school. The VABS-II is completed by the child's primary caregiver and is an individually administered measure of adaptive behavior, especially in those with developmental disorders (48, 49). It can be given from birth to adulthood and is comprised of the following domains: Communication (Receptive, Expressive, Written); Daily Living Skills (Personal, Domestic, Community); Socialization (Interpersonal Relationships, Play and Leisure Time, Coping Skills); Motor Skills (Fine, Gross) and an optional Maladaptive Behavior Index (Internalizing, Externalizing and Other). The VABS-II is utilized to assess personal and social sufficiency with these four major domains.

Behavioral concerns were measured by the Child Behavior Checklist 1½-5 Language Development Survey (CBCL). The CBCL is an instrument used to rate a child's problem behaviors and competencies (50, 51) and was completed by the child's caregiver.

#### Glutathione Measurements

Intracellular reduced, oxidized and total glutathione levels were assessed in blood cells from treatment and control samples collected during weeks 0 (baseline) and week 12 (follow-up). A trained pediatric phlebotomist conducted all blood draw procedures. Blood samples were collected by venipuncture in Vacutainer® CPT™ Mononuclear Cell Preparation Tubes (BD, Franklin Lakes, NJ) with sodium heparin, immediately placed in ice water, and then centrifuged at 2,000 × *g* at 4°C for 10 min. The amounts of total reduced and oxidized glutathione were quantified using a modified Tietze method by Adams (52). Briefly, in this enzyme recycling assay, GSH is oxidized by 5,5′-dithiobis-(2-nitrobenzoic acid) (DTNB) resulting in the formation of GSSG and 5-thio-2-nitrobenzoic acid (TNB). GSSG is then reduced to GSH by glutathione reductase (GR) using reducing equivalent provided by NADPH. The rate of TNB formation is proportional to the sum of GSH and GSSG present in the sample and is determined by measuring the formation of TNB at 412 nm. Standards containing the reduced form of glutathione or the oxidized form of glutathione from 20 to 0.015 uM in 2.5% sulfosalicylic acid were used as standards for calibration curves. The difference in absorbance recorded at 412 nm before and 6 min after the addition of nicotinamide adenine dinucleotide phosphate in the presence of glutathione reductase was utilized to calculate the amount of total glutathione. Oxidized glutathione was quantified in the presence of vinylpyridine and triethanolamine using the same procedure. Reduced glutathione was calculated by subtracting the inactive glutathione concentration from the total glutathione content.

#### Safety Measurements

Any adverse event during the course of the study was monitored and reported to the study staff at week 6 (visit 5) or week 12 (visit 8) in the clinic or directly to the principal investigator throughout the study. Adverse events were considered related to the treatment if they started or worsened following the start of the trial. If they were persistent or severe, the parents were offered the option to discontinue the study.

Additionally, liver and kidney function and cell blood count were assessed in blood and urine at weeks 0 and 12 by a local Quest Laboratories affiliate (Davie, FL). The comprehensive metabolic profile was reviewed by physicians and compared to known reference ranges.

#### Statistical Analysis

Sample size calculations were performed at the beginning of this exploratory trial (*n* = 40). Descriptive statistics were calculated for all study variables. This included means and standard errors of the mean for continuous data and counts and percentages for categorical measures. We assessed the differences in demographic measures between the two groups at baseline using chi-square tests.

The groups were blinded to the statistician for the differences between control and CRWP groups for all variables except for the responders vs. non-responders analysis. A series of mixed, generalized linear models were conducted to look for differences between the CRWP and placebo groups for the physiological assessments. All models included participants' gender, ages, mothers' ages, fathers' ages, and races as covariates. *Post-hoc* tests were conducted using a Bonferroni adjustment. To look for differences in changes between the CRWP and placebo groups for the psychological measurements, a series of paired and unpaired *t*-tests were conducted using a Bonferroni adjustment. Two-tailed tests were performed unless testing a direction hypothesis, in which case a one-tailed test was used. Cohen's D was used to determine the effect size between the two groups. RStudio and R-3.2.2 (53) (https://cran.r-project.org/bin/windows/base/) was used for all statistical analysis, and significance was accepted at *p* < 0.05.

## Results

### Baseline Characteristics

**Figure 2:**
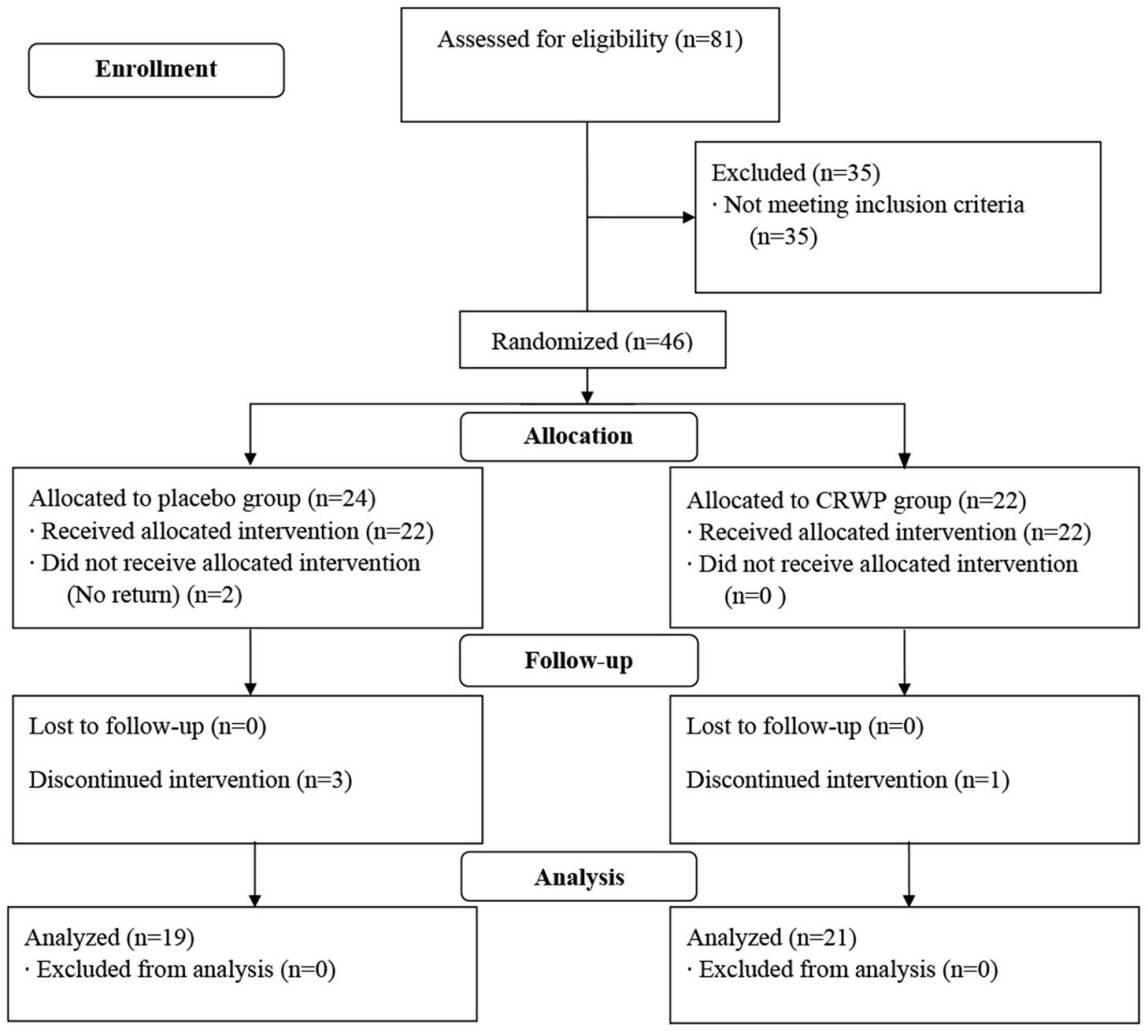
Flow chart of study participant disposition.

A total of 81 participants were screened in this study; 46 were randomized (CRWP, 21; placebo, 24). A total of 40 participants completed the 90-day treatment period (CRWP, 21; placebo, 19). Out of the 24 randomized to placebo, 22 received the allocated intervention, two did not, and three discontinued the before the follow-up visit. The CRWP group received 22 allocations and one discontinued the intervention before the follow-up visit (Figure 2).

At baseline, the participants' demographic and diagnostic characteristics were similar across both groups (Table 1). The average (±SEM) age of participants in both groups was 3.9 (±0.03) and 3.9 (±0.04) years of age in the placebo and CRWP groups, respectively. Most participants were male (placebo = 83% and CRWP = 90%). A wide variety of races were represented in the study, which is indicative of the diversity within the South Florida community. Parental demographic information was also similar across groups, with no significant difference in parents' mean age between groups. However, the percentage of mothers who only graduated from high school was significantly different in the placebo group in comparison to the CRWP group (*p* = 0.04). There were also no differences between the groups at baseline in terms of diagnostic criteria or scores in the ADI-R and ADOS assessments (Table 1). Finally, there were no significant differences in the medications/supplements taken by the placebo or CRWP groups.

**Table 1:** Demographic information for placebo and intervention groups taken at baseline.

**Participant information**			
	**Placebo (*n* = 24)**	**Intervention (*n* = 21)**	***p*-value**
Age (years)	3.9	3.9	0.72
Males, *n* (%)	20 (83)	19 (90)	0.48
**Ethnicity**, ***n*** **(%)**			
Hispanic	5 (21)	10 (48)	0.11
Not Hispanic or Latino	6 (25)	3 (14)	0.37
**Race**, ***n*** **(%)**			
Asian	0 (0)	1 (5)	0.28
Native Hawaiian or other Pacific Islander	1 (4)	0 (0)	0.34
Black or African American	7 (29)	3 (14)	0.23
White	11 (46)	12 (57)	0.45
Other	0 (0)	2 (10)	0.12
**Paternal information**			
Paternal age at child's birth (years)	35.9	36.2	0.77
High school graduate, *n* (%)	3 (14)	6 (32)	0.18
Some college/technical, *n* (%)	4 (19)	5 (26)	0.55
College/professional, *n* (%)	14 (67)	8 (42)	0.18
**Maternal information**			
Maternal age at child's birth (years)	34.1	34.8	0.77
High school graduate, *n* (%)	4 (17)	0 (0)	0.04*
Some college/technical, *n* (%)	3 (13)	4 (20)	0.55
College/professional, *n* (%)	17 (70)	16 (80)	0.69
**Diagnostic characteristics**			
**ADI-R**			
ADI-R (reciprocal social interaction)	12.9 ± 1.54	11.7 ± 1.47	0.41
ADI-R (verbal)	9.11 ± 1.02	10.2 ± 1.31	0.22
ADI-R (non-verbal)	8.40 ± 1.12	9.58 ± 1.16	0.42
ADI-R (restricted behavior)	6.05 ± 0.55	5.26 ± 0.49	0.80
Total	29.00 ± 1.71	26.11 ± 2.19	0.24
**ADOS**			
Composite Score Module 1:	13.5 ± 1.10	14.3 ± 1.92	0.73
Composite Score Module 2:	10.7 ± 1.66	11.2 ± 1.85	0.85

*ADI-R, Autism Diagnostic Interview-Revised; ADOS, Autism Diagnostic Observation Schedule. Values are mean ± SEM*.

Behavioral assessments at baseline were successfully performed in 24 participants in the placebo group and 20 participants within the CRWP group (Table 2). In general, average scores across groups were similar for most behavioral assessments; however, some differences were noted. In the VABS-II, the placebo group had a significantly higher score in daily living skills (*p* = 0.04), coping skills (*p* = 0.02), motor skills (*p* = 0.04), and fine motor skills (*p* = 0.03) in comparison with the CRWP group. Higher scores in the VABS-II indicate more adaptive behaviors, suggesting that the placebo group exhibited less severe clinical manifestations of ASD by this measure. Further, in the Child Behavior Checklist, the CRWP group had higher stress problem T-scores (*p* = 0.04).

**Table 2:** Changes in behavioral assessments in placebo and CRWP group from baseline to follow-up.

**Behavioral assessments**	**Placebo**				**CRWP**				
	**Baseline (*n* = 24)**	**12 weeks (*n* = 21)**	**Δ**	***p*-value**	**Baseline (*n* = 21)**	**12 weeks (*n* = 19)**	**Δ**	***p*-value**	**Baseline placebo vs. Baseline intervention *p*-value**	***p*-value of Δ vs. Δ^†^**
**Childhood autism rating scale**										
**CARS behavior T-score**	40.0 ± 1.87	37.4 ± 1.89	−2.61	0.02^*^	40.2 ± 2.58	38.4 ± 2.83	−1.80	0.04^*^	0.95	0.47
**Preschool language scales (PLS)**										
Total language score	67.3 ± 3.39	66.71 ± 3.68	−0.54	0.46	73.38 ± 4.48	68.80 ± 5.67	−4.58	0.30	0.27	0.17
**Social communication questionnaire (SCQ)**	16.4 ± 1.17	14.4 ± 1.43	−1.99	0.02^*^	16.57 ± 1.12	14.4 ± 1.04	−2.22	0.04^†^	0.41	0.88
**Mullen scales of early learning (MSEL)**										
Early learning composite score	61.0 ± 2.82	63.3 ± 3.84	2.33	0.12	67.1 ± 3.95	68.6 ± 4.53	1.55	0.44	0.20	0.20
**Vineland adaptive behavior scales (VABS-II)**										
Adaptive behavior composite score	82.8 ± 3.41	82.9 ± 3.97	0.13	0.47	71.5 ± 2.85	74.4 ± 3.47	2.85	0.05^†^	0.08	0.03^†^
**Communication domain**	79.9 ± 3.56	79.76 ± 4.41	−0.16	0.45	75.1 ± 4.47	77.2 ± 5.02	2.07	0.05^*^	0.40	0.15
Receptive	10.3 ± 0.66	10.81 ± 0.98	0.56	0.31	9.43 ± 0.80	9.63 ± 0.84	0.20	0.23	0.43	0.84
Expressive	9.38 ± 0.67	9.905 ± 0.69	0.53	0.17	9.38 ± 0.81	9.95 ± 1.09	0.57	0.05^*^	0.87	0.57
Written	15.5 ± 0.86	15.2 ± 0.87	−0.27	0.28	14.48 ± 0.87	14.39 ± 0.80	−0.09	0.17	0.43	0.24
**Daily living skills domain**	82.9 ± 3.42	83.0 ± 3.42	0.12	0.24	74.52 ± 2.99	74.84 ± 3.39	0.32	0.26	0.04^*^	0.25
Personal	11.4 ± 0.76	11.5 ± 0.83	0.10	0.45	9.95 ± 0.55	11.00 ± 0.79	1.05	0.03^†^	0.22	0.05^*^
Domestic	13.5 ± 0.63	13.7 ± 0.56	0.21	0.47	12.10 ± 0.54	11.42 ± 0.50	−0.68	0.37	0.11	0.74
Community	12.2 ± 0. 64	12.3± 0.69	0.16	0.46	10.95 ± 0.54	10.68 ± 0.59	−0.27	0.74	0.16	0.64
**Socialization domain**	79.1 ± 2.30	78.7 ± 3.2	−0.32	0.35	71.24 ± 2.84	73.89 ± 3.69	2.65	0.04^*^	0.06	0.04^*^
Interpersonal	10.4 ± 0.57	10.2 ± 0.79	−0.23	0.26	9.33 ± 0.60	9.632 ± 0.78	0.30	0.27	0.20	0.14
Play and leisure time	10.5 ± 0.58	10.4 ± 0.55	−0.12	0.20	9.33 ± 0.61	9.579 ± 0.70	0.25	0.33	0.20	0.28
Coping skills	13.1 ± 0.47	13.4 ± 0.74	0.27	0.46	11.20 ± 0.50	11.95 ± 0.67	0.75	0.04^*^	0.02^*^	0.23
**Motor skills domain**	86.4 ± 3.33	87.0 ± 4.27	0.57	0.61	78.62 ± 2.85	77.68 ± 2.89	−0.94	0.74	0.04^*^	0.59
Gross	12.7 ± 0.69	12.5 ± 0.84	−0.19	0.83	11.81 ± 0.51	11.16 ± 0.44	−0.65	0.34	0.34	0.88
Fine	12.8 ± 0.63	13.2 ± 0.75	0.35	0.16	11.10 ± 0.61	11.47 ± 0.64	0.37	0.03^*^	0.03^*^	0.63
**Maladaptive behavior domain**	19.2 ± 0.52	19.2 ± 0.55	−0.05	0.47	20.05 ± 0.50	19.44 ± 0.56	−0.61	0.16	0.26	0.04^*^
Internalizing	19.3 ± 0.56	19.5 ± 0.60	0.16	0.17	20.20 ± 0.43	19.53 ± 0.69	−0.67	0.12	0.29	0.02^*^
Externalizing	16.9 ± 0.66	17.0 ± 0.73	0.05	0.99	18.05 ± 0.59	17.58 ± 0.39	−0.47	0.40	0.21	0.48
**Child behavior checklist (CBC)**										
**Total problems**	61.8 ± 2.20	60.3 ± 2.40	−1.50	0.02^*^	62.60 ± 2.00	59.00 ± 2.24	−3.60	0.30	0.82	0.52
**Total scores**										
Internalizing problems T-score	62.4 ± 1.84	60.3 ± 2.17	−2.06	0.04^*^	63.00 ± 1.98	59.40 ± 2.05	−3.60	0.13	0.82	0.95
Externalizing problems T-score	58.8 ± 2.44	57.8 ± 2.24	−1.02	0.16	58.4 ± 1.92	56.4 ± 1.83	−1.97	0.21	0.90	0.91
Stress problems T-score	61.8 ± 2.01	62.3 ± 1.78	0.42	0.41	63.6 ± 2.68	61.7 ± 3.33	−1.90	0.65	0.04^*^	0.38

* *p-value <0.05, two-tail t-test*;

† *p-value <0.05, one-tail t-test*.

*^†^Bonferroni correction applied*.

*CRWP, cysteine-rich whey protein; CARS, Childhood Autism Rating Scale*.

At baseline, the amount of total glutathione, oxidized glutathione, reduced glutathione, and the ratio of oxidized to reduced glutathione was measured in participants' leukocytes. Table 3 shows these baseline measurements for 24 participants in the placebo group and 20 participants in the CRWP group (Table 3). There were no significant differences between groups in any of the glutathione measurements taken at baseline (Table 3).

**Table 3:** Change in glutathione levels from baseline to follow-up in placebo and CRWP groups.

**Glutathione levels: (nM/10^**5**^ WBC)**	**Placebo** **(Mean** **+** **SEM)**				**CRWP** **(Mean** **+** **SEM)**				**Baseline placebo vs. baseline intervention**	***p*-value of Δ vs. Δ**
	**Baseline (*n* = 24)**	**12 weeks (*n* = 16)**	**Δ**	***p*-value**	**Baseline (*n* = 20)**	**12 weeks (*n* = 20)**	**Δ**	***p*-value**		
tGSH	104.2 ± 15.8	88.0 ± 12.8	−16.22	0.50	113.1 ± 16.1	166.7 ± 37.2	53.6	0.17	0.77	0.02^*^
GSSG	10.9 ± 1.57	8.99 ± 1.36	−1.86	0.78	7.95 ± 1.34	12.88 ± 2.70	4.93	0.22	0.14	0.12
GSH	82.5 ± 13.9	66.3 ± 9.8	−16.16	0.53	97.2 ± 15.0	136.0 ± 34.4	38.8	0.47	0.13	0.04^*^

* *p-value <0.05*.

*CRWP, cysteine-rich whey protein; WBC, white blood cells; SEM, standard error of the mean; tGSH, total glutathione; GSSG, glutathione (inactive, oxidized form); GSH, glutathione (active, reduced form). Values are mean ± SEM*.

### Behavioral Assessments and Glutathione Measurements

Changes in the different behavioral assessments used in this study: CARS, PLS, SCQ, Mullen, VABS-II and CBCL for both groups are depicted on Table 2. When comparing baseline to follow-up changes in the CRWP (*n* = 21) and placebo (*n* = 19) groups, behavioral improvements were seen in both groups; however, the CRWP group improved in more areas than the placebo group.

When comparing changes between baseline and follow-up in the two groups (Δ vs. Δ), no significant differences were observed after 3 months in the CARS, PLS, SCQ or CBC behavioral scales (Table 2). However, the parent-rated behavioral assessment VABS-II demonstrated significant improvements in the CRWP group compared to the placebo group in the composite score (effect size 0.98; 95% CI 1.42–4.02; *p* = 0.03). Also, significant changes were observed in multiple domains/sub-domains representing different aspects of adaptive behaviors associated with ASD symptoms in the intervention group. Specifically, the socialization domain (effect size 1.07; 95% CI 1.82–4.28; *p* = 0.04), domestic daily living skills (effect size 0.73; 95% CI 0.34–1.55; *p* = 0.05), maladaptive behavior domain (effect size 0.54; 95% CI −1.12 to 0.10; *p* = 0.04), and internalizing subdomain (effect size 0.73; 95% CI −1.40 to 0.34; *p* = 0.02) all showed significant improvements with the CRWP supplementation after 3 months.

**Figure 3:**
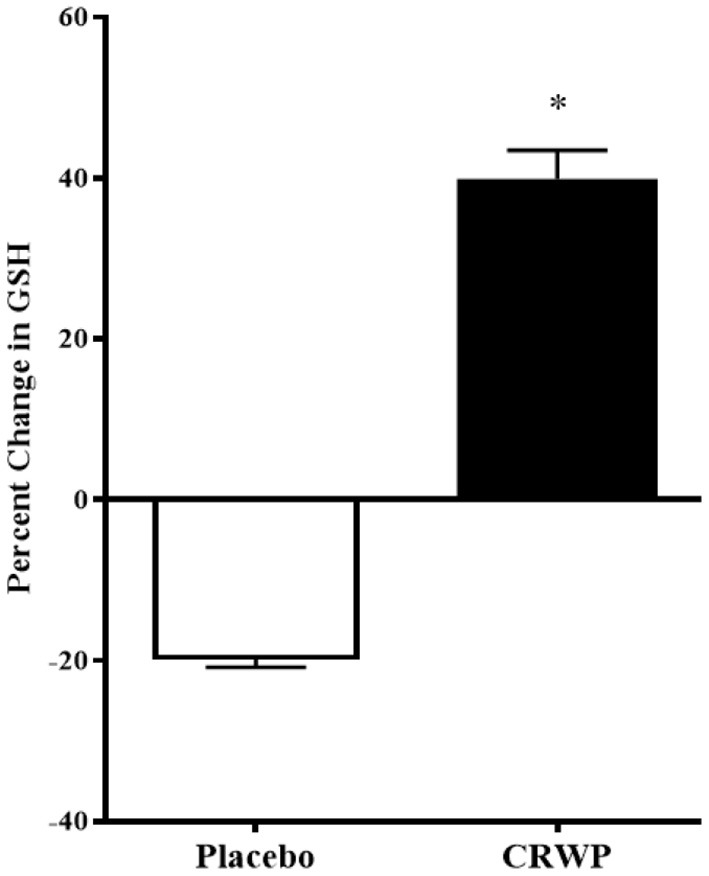
Percent change from baseline of reduced glutathione levels in placebo and CRWP groups. *p < 0.05. CRWP, cysteine-rich whey protein.

Glutathione levels were successfully assessed in 16 participants in the placebo group and 20 participants within the CRWP group at the end of the study (Table 3). Higher increases of total and reduced glutathione were observed from baseline to follow-up in the CRWP group (Figure 3). In contrast, no significant changes in total, reduced or oxidized glutathione levels were observed in the placebo group (Table 3). After the 90-day supplementation, changes in both total (*p* = 0.02) and reduced glutathione (*p* = 0.04) in the CRWP group were significantly higher than changes in the placebo group (Table 3; Figure 3). However, behavior improvements observed using the VABS-II were not significantly correlated with changes in glutathione levels.

### Sub-analysis of the CRWP Group

**Figure 4:**
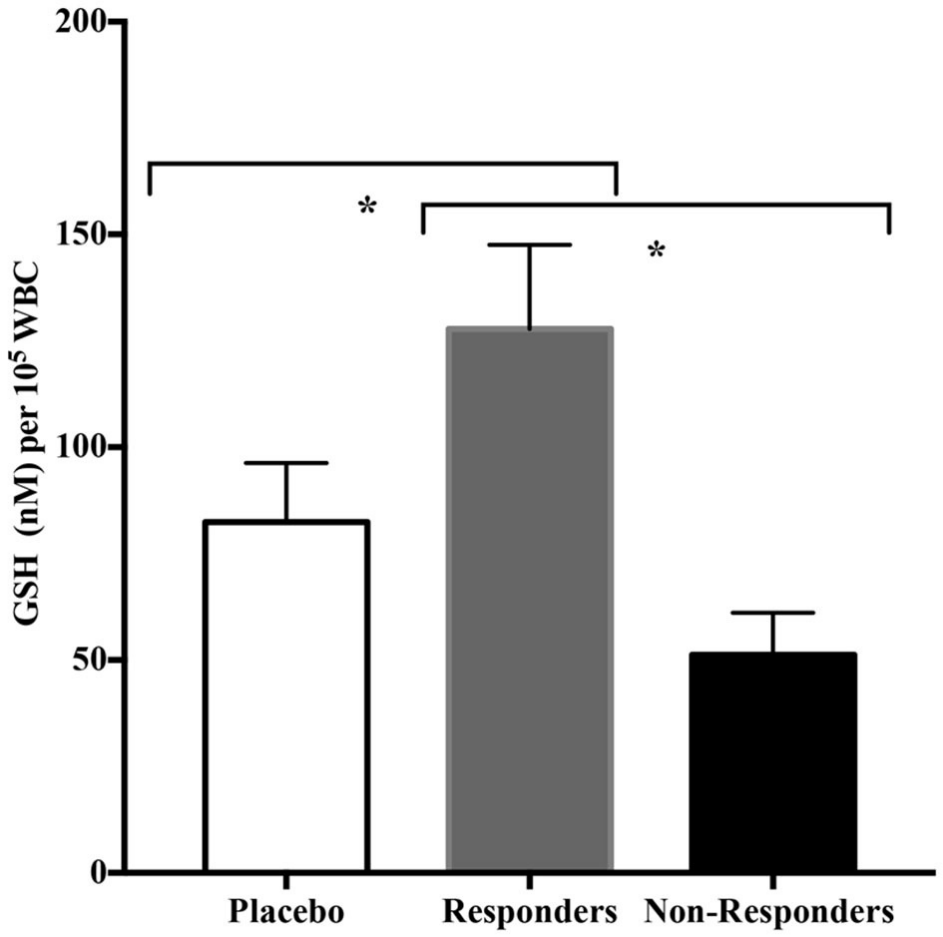
Baseline glutathione levels in placebo, responder and non-responder groups. *p < 0.05.

Because there were a significant number of participants in the CRWP group in whom significant improvements in adaptive behavior were observed using the VABS-II, we explored the common characteristics in this group of responders. The objective of this sub-analysis was to identify common characteristics in the responders to the CRWP supplementation using the VABS-II assessment. Changes of >2 points or 1 SD in VABS-II composite scores were used to discriminate “responders.” Out of the 19 who completed the behavioral assessments in the CRWP group, 12 were identified as responders vs. only 5 out of the 21 who completed behavior assessments in the placebo group (*p* = 0.03). When comparing the responders from the CRWP group (*n* = 12) to the non-responders in the same group (*n* = 7), there was a significant difference in age with the responders being significantly older (4.23 ± 0.22 years) than non-responders (3.48 ± 0.18 years; *p* = 0.03). Moreover, the responders had significantly higher levels of total glutathione (*p* = 0.01) and reduced glutathione (*p* = 0.01) at baseline compared to the non-responders (Figure 4) within this group. However, no difference was found between the two groups when comparing changes in total glutathione levels (responders *p* = 0.05, non-responders *p* = 0.06) (Table 4).

**Table 4:** Demographics and glutathione levels of responders (*n* = 12) and non-responders (*n* = 7).

**Characteristics of responders and non-responders in the CRWP group**	**Responders (*n* = 12)**	**Non-responders (*n* = 7)**	***p*-value**
Age (mean + SEM)	4.23 ± 0.22	3.48 ± 0.18	0.03^*^
**Parent information (mean** **+** **SEM)**			
Paternal age at child's birth (years)	34.3 ± 3.84	39.5 ± 5.2	0.45
Maternal age at child's birth (years)	33.1 ± 2.47	36.9 ± 3.0	0.35
**Baseline glutathione levels**			
tGSH (uM/10^5^ WBC)	144.8 ± 21.4	65.6 ± 11.7	0.01^*^
GSH (uM/10^5^ WBC)	127.8 ± 19.8	51.27 ± 9.9	0.01^*^
**Change in glutathione**			
tGSH (uM/10^5^ WBC)	4.60 ± 6.7	6.49 ± 4.0	0.83
GSH (uM/10^5^ WBC)	3.68 ± 5.6	4.17 ± 3.7	0.95

* *Significant difference between placebo and non-responder subgroups where p <0.05*.

*CRWP, cysteine-rich whey protein; SEM, standard error of the mean; WBC, white blood cells; GSH, glutathione (active, reduced form); GSSG, glutathione (inactive, oxidized form); tGSH, total glutathione*.

**Figure 5:**
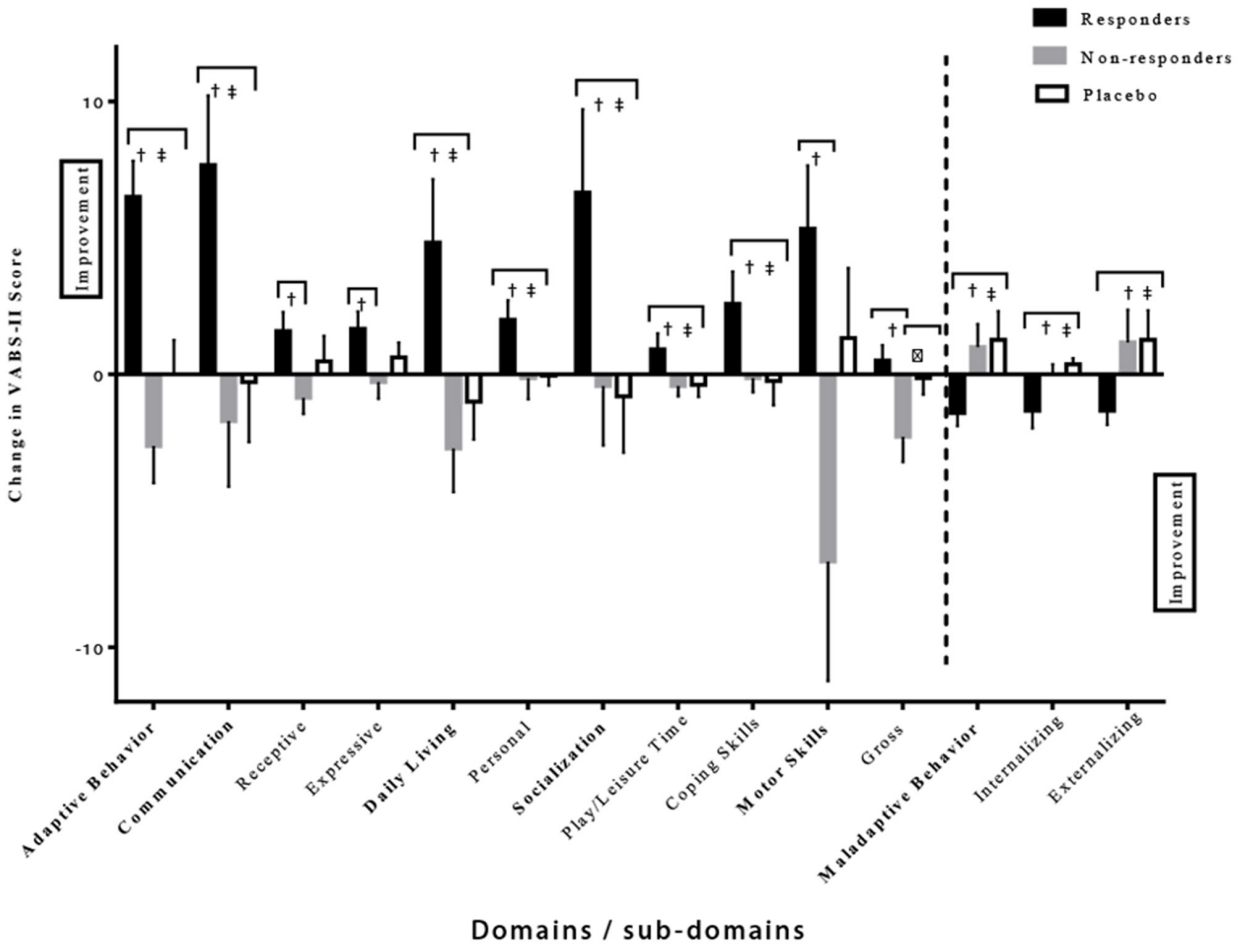
Significant differences in various domains/sub-domains of VABS-II in the responder, non-responder and placebo groups. ^†^Significant difference between responder and non-responder groups. ^‡^Significant difference between responder and placebo groups. ^δ^Significant difference between non-responder and placebo groups. One-tail *t*-tests were utilized with significance reached at *p* < 0.05. VABS-II, Vineland Adaptive Behavior Scale, 2nd edition.

Further analysis of the VABS-II was used to compare the behavioral changes in the responders vs. the non-responders in the intervention group (Figure 5). The responders showed a much larger improvement in the given domains/sub-domains of the VABS-II, including the overall adaptive behavior composite score (*p* < 0.0001 and *p* = 0.0003 compared to non-responders and placebo, respectively). In addition, differences were noted in the communication domain score (*p* = 0.008 and *p* = 0.01 compared to non-responders and placebo, respectively), with differences noted in the receptive V-scale score (*p* = 0.01 compared to non-responders), and the expressive v-scale score (*p* = 0.03 compared to non-responders); in the daily living skills domain score (*p* = 0.008 and *p* = 0.007 compared to non-responders and placebo, respectively) with differences observed in the personal v-scale score (*p* = 0.03 and *p* = 0.004 compared to non-responders and placebo, respectively). Responder improvements were also noted in socialization domain score (*p* = 0.04 and *p* = 0.02 compared to non-responders and placebo, respectively), with significant differences in play and leisure time (*p* = 0.03 and, *p* = 0.04 compared to non-responders and placebo, respectively), coping skills (*p* = 0.03 and *p* = 0.04 compared to non-responders and placebo, respectively), and in the motor skills domain score (*p* = 0.02 compared to non-responders) including the gross motor score (*p* = 0.01 compared to non-responders). Moreover, decreases in internalizing (*p* = 0.04 and *p* = 0.0003 compared to non-responders and placebo, respectively), externalizing (*p* = 0.02 and *p* = 0.008 compared to non-responders and placebo, respectively), and maladaptive behavior scores (*p* = 0.01 and *p* = 0.003 compared to non-responders and placebo, respectively) were also seen in the responders' group (Table 5). It is important to highlight that no differences were found in baseline composite VABS-II scores between responders and non-responders (*p* = 0.24). These results show a favorable effect of CRWP on a range of behavior measures in those that responded to this intervention.

**Table 5:** Changes in VABS-II scores in responders, non-responders, and placebo groups.

**Vineland adaptive behavior scale**	**Responders (*n* = 12)**	**Non-responders (*n* = 7)**	**Placebo (*n* = 21)**	***P*-value responders vs. non-responders**	***P*-value responders vs. placebo**
**Adaptive behavior composite standard score**	6.5 ± 1.32	−2.86 ± 0.91	0.00 ± 1.25	<0.0001^†^	0.0003^†^
**Communication standard total score**	7.67 ± 2.55	−1.71 ± 2.40	−0.29 ± 2.20	0.008^†^	0.01^†^
Receptive V-scale score	1.58 ± 0.69	−0.86 ± 0.59	0.48 ± 0.93	0.01^†^	0.21
Expressive V-scale score	1.67 ± 0.63	−0.29 ± 0.60	0.62 ± 0.54	0.03^†^	0.08
Written V-scale score	0.64 ± 0.47	0.29 ± 0.52	−0.29 ± 0.49	0.32	0.12
**Daily living skills standard total score**	4.83 ± 2.31	−2.7 ± 1.6	−1.00 ± 1.39	0.008^†^	0.007^†^
Personal V-scale score	2 ± 0.71	−0.14 ± 0.77	−0.05 ± 0.36	0.03^†^	0.004^†^
Domestic V-scale score	−0.08 ± 0.57	−0.71 ± 0.60	0.05 ± 0.63	0.23	0.45
Community V-scale score	0.33 ± 0.45	−0.43 ± 0.62	−0.05 ± 0.51	0.19	0.20
**Socialization standard total score**	6.67 ± 3.04	−0.43 ± 2.17	−0.81 ± 2.06	0.04^†^	0.02^†^
Interpersonal relationships V-scale score	0.83 ± 0.82	0.29 ± 0.57	−0.38 ± 0.59	0.30	0.12
Play and leisure time V-scale score	0.92 ± 0.58	−0.43 ± 0.37	−0.38 ± 0.44	0.03^†^	0.04^†^
Coping skills V-scale score	2.58 ± 1.18	−0.15 ± 0.51	−0.24 ± 0.89	0.03^†^	0.04^†^
**Motor skills standard total score**	5.33 ± 2.32	−6.86 ± 4.4	1.33 ± 2.56	0.02^†^	0.05
Gross V-scale score	0.5 ± 0.57	−2.29 ± 0.92	−0.14 ± 0.60^δ^	0.01^†^	0.09
Fine V-scale score	1.33 ± 0.54	0 ± 0.62	0.57 ± 0.39	0.07	0.13
**Maladaptive behavior total v-scale score**	−1.42 ± 0.47	1.0 ± 0.84	1.26 ± 1.05	0.01^†^	0.003^†^
Internalizing V-scale score	−1.33 ± 0.64	0 ± 0.37	0.37 ± 0.22	0.04^†^	0.0003^†^
Externalizing V-scale score	−1.33 ± 0.51	1.17 ± 1.20	1.26 ± 1.08	0.02^†^	0.008^†^

† *p <0.05, one-tail t-test*.

δ *Significant difference between placebo and non-responder subgroup where p <0.05*.

*VABS-II, Vineland Adaptive Behavior Scale, 2nd edition. Values are mean ± SEM*.

### Safety

There were no serious adverse events reported in either treatment group. Table 6 details all adverse events reported throughout this study. Compliance was assessed by the weight of the canisters before and after treatments and did not show significant differences between the groups (90.5% used in the placebo group vs. 89.9% in the CRWP group; *p* = 0.91). There were more dropouts in the placebo group compared to the CRWP group. According to parents' diaries and records from visit 5, nausea was mostly reported in the first weeks of the trial in each group but tended to improve as parents adapted their technique to reconstitute the supplement powder with different juices/meals. There were also no significant changes in any of the complete blood count and comprehensive metabolic panel values obtained throughout the study.

**Table 6:** Adverse events or acute health complications reported throughout the study.

**Adverse events, *N* (%):**	**Placebo (*n* = 19)**	**Intervention (*n* = 21)**	***p*-value**
Bronchitis/cough/respiratory infection	2 (8)	4 (19)	0.45
Cold symptoms	7 (29)	9 (43)	0.70
Constipation	3 (13)	1 (5)	0.25
Diarrhea	1 (4)	4 (19)	0.19
Emesis/nausea	4 (21)	7 (33)	0.39
Fever	2 (8)	4 (19)	0.45
Rash	0 (0)	1 (5)	0.34
Other/etc.	3 (13)	4 (19)	0.77

## Discussion

In this study, we sought to determine if supplementation with CRWP would improve behaviors and intracellular glutathione levels in children with ASD aged 3–5 years old. We utilized a comprehensive behavioral assessment to explore the impact of this supplementation, making this approach unique in evaluating multiple behavioral aspects in this condition. Furthermore, we used a rice protein powder, which mimics the amount of protein obtained from whey, as a placebo. Although several of the behavioral scales did not showed significant differences when comparing changes between the two groups from baseline to follow-up, the VABS-II demonstrated a favorable effect of this supplement on several aspects of ASD behavior. Further, an improvement in antioxidant capacity was demonstrated by increased glutathione levels.

Significant improvements were observed in both groups when comparing baseline to follow-up behavioral scores assessments in some tests. It was expected that all children participating in this study would show some behavioral improvement, particularly because they had access to a high standard of care during the study consisting of preschool centers and therapies that provide special services to this population. However, when comparing the magnitude of changes between the two groups after the 3-month trial period (Δ vs. Δ), significant behavioral improvements with medium-large effect sizes were seen in the children's behavioral adaptive skills as measured by the VABS-II in the CRWP group. Therefore, only randomized, double-blind, placebo-controlled studies can reveal accurate improvements with specific interventions. It is worth noting that children within the intervention group had to improve more than the placebo group for significant outcomes to be observed since their baseline values indicated they were more severely affected by the disorder at the start of the trial.

In children with ASD, several behavioral improvements have been associated with nutritional interventions. NAC supplementation was associated with a decrease in irritability using the Aberrant Behavior Checklist ABC and in repetitive behaviors using the Repetitive Behavior Scale-Revised (RBS-R) and Social Responsiveness Scale (SRS) assessments in a pilot study (23). In contrast, Wink et al. (24) found no behavioral improvements with a similar study design. Vitamin B6 supplementation was associated with positive changes in sleep and gastrointestinal issues in a randomized, double-blind, placebo-controlled 3-month study in 20 children with ASD using a parent-rated scale (25). Other supplements closely related to the transmethylation/transsulfuration pathway were also associated with improved motor skills in a case study (54) and multiple domains of the behavioral assessments (26) when focusing on a subgroup of participants. The approach taken to distinguish a subgroup of children who better responded to a nutritional intervention in terms of behavior, is comparable to the present study. Additionally, our study mirrored the results of another research (28), which showed significant increases in many of the same VABS-II scores after supplementation with methylcobalamin plus folinic acid. Each study utilized a clinician, parent, or a combination of scales to assess changes in behavior. Different studies use a variety of diverse scales and study designs to assess behavioral changes, making it very difficult to compare our results with most previous studies using other nutritional interventions.

A total of 12 out of 20 (60%) children were recognized as responders to the intervention due to the >2-point improvement in VABS-II overall adaptive composite score. Recently, Chatham et al. (55) showed that the minimal clinically significant difference in children with ASD ranged from 2 to 3.75 points, supporting our approach to identifying these children as responders. Moreover, the fact that parents blinded to the intervention reported significant differences in their child's adaptive behavior (VABS-II) on scales assessing several affected domains plus daily living activities, is compelling. Improvements in the VABS-II of 4-5 points were also noted in the Phase II clinical trial of balovaptan in autistic adults. Although measuring adaptive behavior was not a primary outcome of that trial, the improvements in this assessment gained the FDA's breakthrough therapy label (56). Because ASD encompasses a broad phenotype in terms of its behavioral presentation without a known etiology, it is expected that not all patients will respond equally to one intervention. Therefore, children diagnosed with ASD and impaired behavior in areas that demonstrated significant improvements with this intervention may be good candidates for this nutritional supplementation. Further studies will be needed to confirm these preliminary results and test this supplement in older individuals with this condition.

In fact, others have also found that targeting antioxidant capacity using different interventions such as methylcobalamin, folinic acid (28, 30) or n-acetylcysteine (23, 57) may be more beneficial in a subgroup of children with this condition. Up to date, there are no genotypes or phenotypes associated with responders to these interventions; however, clinicians and caregivers will significantly benefit from identifying patients that may potentially respond to these treatments. It is also possible that additive beneficial effects may be found when combining these therapies as in the case of methylcobalamin plus folinic acid (28, 30).

Significant improvements in the glutathione levels of children with ASD were also confirmed in this study using a nutritional approach known to increase glutathione biosynthesis. As expected, children assigned to the supplement experienced a 40% increase in the reduced form of glutathione because this supplement provides a natural source of cysteine, the rate-limiting step in glutathione biosynthesis, that ultimately leads to increased intracellular levels.

Other interventions have demonstrated comparable glutathione increases to those in our CRWP group. The combination of methylcobalamin and folinic acid induced increases of 15% in total glutathione and 20% in reduced glutathione, respectively (28), while NAC treatment has demonstrated a 60% increase from baseline (24). The importance of targeting glutathione levels is relevant because significant differences in glutathione and its related metabolites have been found in plasma (16, 17), white blood cells (58, 59), and post-mortem brains (11, 21) of participants with ASD. This finding is also supported by several genetic variations seen in ASD patients related to the transmethylation/transsulfuration pathway, where glutathione is one of the byproducts (16–18). The hypothesis that low glutathione levels are related to ASD symptoms has been partially validated because several treatments targeting this deficiency have been proven to be efficacious at modifying behavioral symptoms in children with this condition (29).

We then investigated the correlation between behavioral improvements and changes in intracellular glutathione concentrations. Although it could be anticipated that the magnitude of glutathione increases would correlate with behavioral improvements as in other studies (23, 28), we found no correlation between changes in glutathione levels and improvement in VABS-II scores. However, both core areas of ASD behaviors and antioxidant capacity were positively impacted by this intervention. It is also possible that the benefit of the CRWP may not be limited to its efficacy at increasing antioxidant capacity, but its ability to improve overall health.

When examining the relationship between baseline glutathione levels, the responders (*n* = 12) had significantly higher baseline concentrations of both total and reduced glutathione than baseline and non-responders (*n* = 8). This suggests that these participants were closer to obtaining a threshold in their glutathione levels leading to positive changes in behavior. It is possible that children with lower baseline glutathione levels may need a higher dose or a longer intervention to attain similar behavioral outcomes.

Evidence-based effective and safe interventions in ASD are needed to ease some of the behavioral challenges seen in this condition. The use of complementary and alternative medicine has been reported to be around 74% in children with ASD (60–63). By avoiding established pharmacological treatments that may produce significant side effects, parents try to alleviate behavioral problems and associated comorbid conditions using alternative treatments. Therefore, there is a significant need to investigate the efficacy of complementary and alternative therapies and their tolerability in children with ASD while also identifying those that respond to these interventions.

The limitations of this exploratory study include the small sample size, narrow age sample and short treatment intervention. However, we feel that our robust study design achieved our study goals in a challenging, well-defined population of preschool children. Future studies including a broader age group and using specific behavioral assessments and scales are warranted. It would also be pertinent to include a cohort of neurotypical children in a similar study to compare any changes in glutathione levels and behaviors in that population with those of children with ASD. Our study also featured predominantly male children. It would be interesting to investigate a more extensive study population to assess the potential beneficial effects of CRWP in female children with ASD, as the manifestations of ASD can differ between males and females (64).

## Conclusion

In conclusion, this study demonstrated that nutritional intervention with CRWP effectively improved glutathione levels in children with ASD, ameliorated some behavioral domains impacted in ASD and was well-tolerated. Future studies with specific outcomes, larger sample sizes, different ages, taking into consideration baseline glutathione levels, are needed to better assess treatment outcomes. If further evidence for a positive effect is revealed, supplements such as CRWP could be recommended to help support the behavioral aspects of ASD.

## Data Availability Statement

The datasets presented in this article are not readily available because they belong to Nova Southeastern University and Immunotec Inc. and can only be available upon request to the PI after approval from the funding sources. Requests to access the datasets should be directed to Ana Maria Castejon, castejon@nova.edu.

## Ethics Statement

The studies involving human participants were reviewed and approved by Institutional Review Board at Nova Southeastern University in Fort Lauderdale, FL, USA. Written informed consent to participate in this study was provided by the participants' legal guardian/next of kin.

## Author Contributions

AC, NS, and SK contributed to conception and design of the study. JS and AS organized the database and conducted GSH measurements. IR contributed with good practices of the clinical trial regulations. RF and EP contributed with clinical measurements. PH performed the statistical analysis. AC and JS wrote the first draft of the manuscript. NS and SK wrote sections of the manuscript. All authors contributed to manuscript revision, read, and approved the submitted version.

## Funding

This study was supported in part by Immunotec Inc. and Nova Southeastern University. The funders had no role in the design of the study, in the collection, analysis, and interpretation of data, or in writing the manuscript.

## Conflict of Interest

The authors declare that the research was conducted in the absence of any commercial or financial relationships that could be construed as a potential conflict of interest.

## Publisher's Note

All claims expressed in this article are solely those of the authors and do not necessarily represent those of their affiliated organizations, or those of the publisher, the editors and the reviewers. Any product that may be evaluated in this article, or claim that may be made by its manufacturer, is not guaranteed or endorsed by the publisher.

## Abbreviations

ADI-R: Autism Diagnostic Interview-Revised
ADOS: Autism Diagnostic Observation Schedule
ASD: Autism Spectrum Disorder
CARS: Childhood Autism Rating Scale
CBC: Complete blood count
CBCL: Childhood Behavior Checklist
CMP: Comprehensive metabolic panel
CRWP: Cysteine-rich whey protein
GSH, Glutathione (active: reduced form)
GSSG, Glutathione (inactive: oxidized form)
MSEL: Mullen Scales of Early Learning
NAC: N-acetylcysteine
PLS-5: Pre-school Language Scale- 5th edition
ROS: Reactive oxygen species
RRBI: Restricted and repetitive behaviors and interests
SAM: S-adenosylmethionine
SAH: S-adenosylhomocysteine
SCQ: Social Communication Questionnaire
tGSH: Total glutathione (reduced + oxidized)
VABS-II, Vineland Adaptive Behavior Scale: 2nd edition.
